# Long-term observation of amphibian populations inhabiting urban and forested areas in Yekaterinburg, Russia

**DOI:** 10.1038/sdata.2015.18

**Published:** 2015-05-12

**Authors:** Vladimir L. Vershinin, Svetlana D. Vershinina, Dmitry L. Berzin, Darya V. Zmeeva, Alexander V. Kinev

**Affiliations:** 1 Institute of Plant and Animal Ecology, Ural Branch of Russian Academy of Sciences, 8 Marta 202, Yekaterinburg 620144, Russia; 2 Eltsyn Ural Federal University, Mira 19, Yekaterinburg 620002, Russia; 3 Creative Scientist, Inc., Durham, NC 27713, USA

**Keywords:** Urban ecology, Herpetology

## Abstract

This article presents data derived from a 36 year-long uninterrupted observational study of amphibian populations living in the city and vicinity of Yekaterinburg, Russia. This area is inhabited by six amphibian species. Based on a degree of anthropogenic transformation, the urban territory is divided into five highly mosaic zones characterized by vegetation, temperature, and a distinctive water pollution profile. Population data is presented year-by-year for the number of animals, sex ratio, and species-specific fecundity including the number and quality of spawns for the following amphibian species: *Salamandrella keyserligii, Rana arvalis, R. temporaria, Lissotriton vulgaris*, and *Pelophylax ridibundus*. These data provide an excellent opportunity to assess an urban environment from an animal population-wide perspective, as well as revealing the forces driving animal adaptation to the anthropogenic transformation of habitats.

## Background and Summary

**Figure 1:**
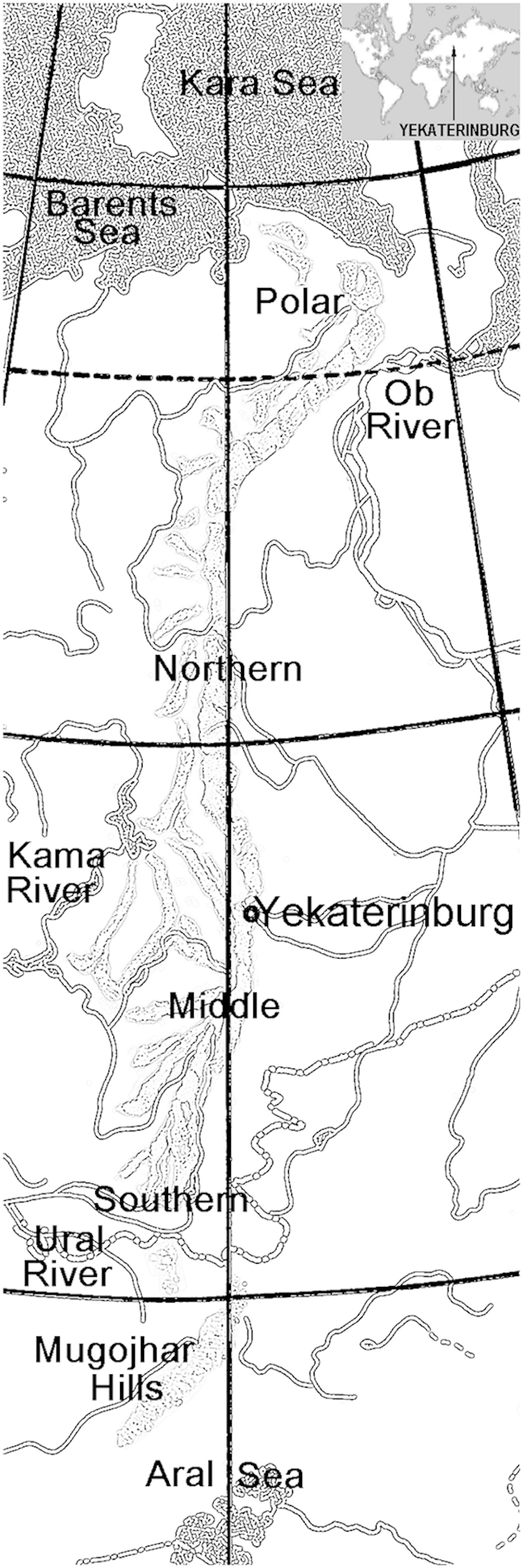
Map of the Ural Region.

In the history of the biosphere, urbanization is a recent factor and therefore, its long-term effects on animal populations are largely unexplored. A city is a special type of environment where animals are exposed to a complex mixture of pollutants, often acting synergistically, and it is difficult to forecast the long-term consequences of such a synergism^1,2^. Previous work suggested that since amphibian development is environmentally sensitive, changes in urban amphibians at individual and/or population level could serve as an indication of environmental pollution^3^. Accordingly, many studies tried to analyze how urbanization affects various aspects of amphibian biology. However, published data usually lacks comprehensiveness^4^ or spans only short periods. To evaluate the long-term effects of urbanization, we are conducting a multi-year comprehensive ecological and population-wide study of amphibians inhabiting Yekaterinburg, one of the largest Russian industrial centers located in Ural Mountains (Figure 1).

**Figure 2:**
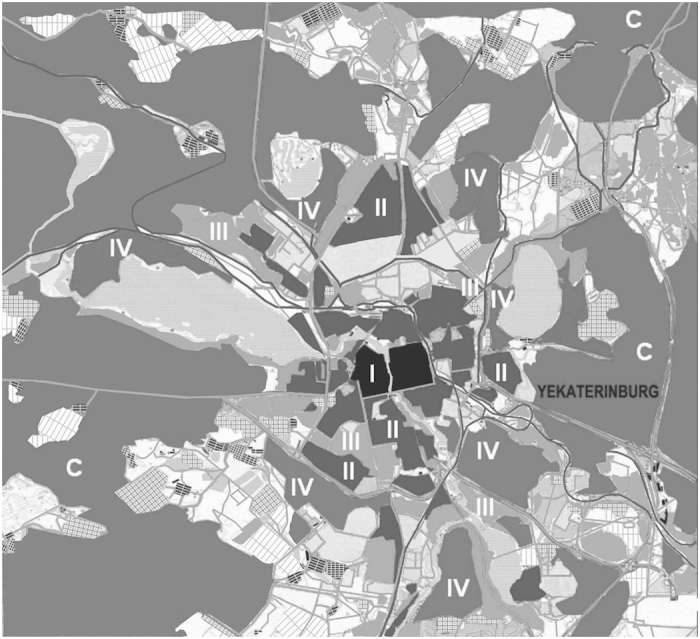
Yekaterinburg’s landscape typification. Zones (see text for details): I—city center; II—multistory buildings; III—low-rise buildings, residential areas; IV—parks and recreation areas; and C—forests.

Yekaterinburg is a typical urban territory with a well-developed city infrastructure, dense human population, and a long history of industrial pollution. The city’s landscape can be divided into 5 zones reflecting various levels of anthropogenic transformation^5^. Each zone is characterized by a particular vegetation community, water chemistry profile and temperature regime (see Vegetation_description_eng.csv and Temperature_April_May_eng.csv (Data Citation 1). Further details are presented below in Landscape typification section. Zone I is the most transformed area lacking open water sources and since amphibian development strictly depends on water, no amphibian species is found there. The distribution of amphibians in other urban areas is remarkably discontinuous because roads, buildings and other artificial barriers efficiently separate individual habitats (Figure 2). The urban Zones II-IV and the forested area around the city (Zone C, native environment) are inhabited by the following five amphibian species (listed in order from the most to the least abundant): *Rana arvalis*, *Lissotriton vulgaris, Pelophylax ridibundus, Salamandrella keyserlingii*, and *R. temporaria* (see Populations_status_1977_2013_eng.csv (Data Citation 1)). The sixth species, *Bufo bufo* only occasionally appears, in Zone C and therefore, no data is shown.

In the course of our study, we monitored the number of eggs (and, in some cases, their size) and the number of adult females through number of spawns (see corresponding files on fecundity, egg size, number of spawns, and populations’ status). Importantly, extinction of amphibian populations resulted from habitat destruction has been recorded at ~40% of sites. Thus, *S. keyserlingii* has disappeared from one site, *L. vulgaris*—from seven, *R. arvalis*—from eleven, *R. temporaria* and *P. ridibundus*—from five each. Of note, *P. ridibundus* has also appeared at five new urban and some natural sites thus demonstrating that this species is currently actively expanding in the Ural region^6^. See also the file on these populations’ statuses.

Since amphibians spawn and develop in ponds, we regularly test the quality of water sources (see Water_1980_2013_basic_eng.csv (Data Citation 1)). There are environmental gradients between zones that are quite steep and remarkably stable over time including differences in pH, temperature, and the level of pollution. For example, ponds located in Zones II-IV have average pH 7.3, while ponds in Zone C have average pH 6.6. Higher chemical oxygen demand and mineral content is characteristic of ponds located in most urbanized Zones II and III compared to those in recreational Zone IV or forest areas (Zone C). In addition, average water temperatures are approximately 2–3 °C higher in ponds in Zones II-IV than in Zone C, although temperatures in Zone IV have increased only during the last decade (Tables 1 and 2 and Temperature_April_May_eng.csv (Data Citation 1)).

During our period of observation, *R. temporaria* has significantly declined in Zone III and completely disappeared from Zone II. Contrary to that, *P. ridibundus* has emerged in Zone IV (see Populations_status_1977_2013_eng.csv (Data Citation 1)). Apparently, *P. ridibundus* is an ecologically malleable species and, thus, not only prevails in natural ecosystems but also can successfully reproduce in urban areas. Similarly, *L. vulgaris* usually reproduces successfully in Zone III, although it is absent in the most transformed sites in Zone II. Both species belong to an evolutionary younger taxon compared to *B. bufo* and *S. keyserlingii*, both of which belong to older taxonomic groups. Neither of the latter species perform well in the urban environment. Thus, *B. bufo* can never be found within the city and *S. keyserlingii* is absent from Zone II and is very rare in Zone III and in some of the most transformed places in Zone IV (see Populations_status_1977_2013_eng.csv (Data Citation 1)).

We suggest that the dynamics of amphibian populations in an urban environment is associated with ecological plasticity of the species, which in turn seems to relate to their evolutionary origin. Thus, polymorphic species belonging to evolutionary younger taxons have a survival advantage in an urban environment.

## Methods

### Geographic location

Yekaterinburg is situated on the eastern side of the central part of the Ural Mountains. The Urals are a natural barrier between European and Asian plant and animal populations and are characterized by a considerable heterogeneity of landscapes including forested hills, lakes, and agricultural land. The area is characterized by a continental climate with four winter months (October—February) and three months of summer (June—August). Average winter temperature is −16 °C (average high +6, average low −18) and average summer temperature is +20 °C (average high +23, average low +10).

### Collection sites

The material has been collected at 26 locations in the city and vicinity of Yekaterinburg (Table 3) See also Habitats_coordinates.csv (Data Citation 1).

Every spring (1977–2013) 55 ponds were searched by the authors for an assessment of spawn numbers. In summer and autumn every habitat mentioned above was searched for adults and juveniles. Both procedures were carried out at least four times during the season.

### Landscape typification

Urban landscape was typified based on land use and other results of human activity, such as height and concentration of buildings, human population density, level of pollution, types of vegetation, etc.^5^ Closely placed multistory buildings and most of the ground covered by asphalt and concrete characterize Zone I (city center), which also has the highest human population density. Since there are no ponds or open springs, no amphibians live in Zone I. Although dense multistory development and compact human population are also characteristic to Zone II, it has some open soils and water streams as well as small ponds, which amphibians are able to use for spawning and development. Amphibian habitats in Zone II are isolated from each other by roads, long buildings, and other infrastructural elements. Zone III has lower human population density and a prevalence of low-rise building. There are many open grounds in gardens and city parks and a variety of small ponds, springs, and rivers, thus, providing amphibians with many opportunities to breed and grow. Zone IV is the least polluted urban territory as it is primarily an area of parks and recreation that are connected to forests around the city. Zone IV also has the lowest human population and a low industrial presence. Finally, Zone C (control) is comprised of the forests surrounding the city. Samples from Zone C were collected at a site located 23 km from the city line, which we consider a natural environment for amphibian populations.

### Morphological measurements

Morphological measurements (snout-vent body length of specimens) have been made by manual caliper from 1977 to 2002. After that, a digital caliper from (Kraftool, Germany) has been used. Both calipers have the same scale interval −0.01 mm. Measurement of body, liver and heart mass was performed on a digital and torsion balance (Shimadzu) with a scale of 0.2 mg. Throughout the entire observation, we used the same volumetric laboratory glassware (Yugoslavia) and binocular microscope (LOMO, Russia). Methodology on the survey techniques are presented below (Fecundity and Eggs collection methods section).

### Quality of water

Water samples were collected twice a season: at the end of spawning and at the peak of metamorphosis. Sample volume was 1.5 liters. File Water_1980_2013_basic_eng.csv (Data Citation 1) contains data on quality of water in spawning ponds in 1980–2013, which was an important part of landscape typification. In the city, amphibians reproduce in small ponds, which are closed water bodies that accumulate pollutants washed in from the terrain and surrounding roads, brought in by atmospheric precipitation and dumped in by businesses and households. Water was sampled in springs at the end of the spawning and again in the summer at the time of metamorphosis. Dr Galina Oboldina at the Institute of Complex Use and Protection of Water Resources in Yekaterinburg analyzed water in 1980-81 and 1987–1989. Dr Tatiana Yeremkina at the Urals Research Institute of Aquatic Biological Resources and Aquaculture, Yekaterinburg analyzed samples in 2003–2008. Finally, Dr Nina Penkina at the Laboratory of Physical and Chemical Analyzes, Ural State Mining University, Yekaterinburg analyzed the samples in 2009–2013. The following water parameters are shown:

- Acidity (pH);
- Mineralization—an integrated parameter of general level of inorganic substances in water;
- Chlorides—an indirect measurement of inorganic substances brought in from the terrain;
- Sulfates—an indirect measurement of inorganic substances originating from the air;
- Oxidisability—a measurement of total level of organic matter (carbon) in water samples;
- Biochemical Oxygen Demand (BOD, mg/dm^3^)—an indicator of organic water pollution (level of eutrophication), which is measured as the amount of dissolved oxygen necessary to break down organic material present in water by aerobic biological organisms;
- Chemical Oxygen Demand (COD, mg/dm^3^)—an indicator of the total level of oxidizable matter in water including both organic and inorganic substances.

### Temperature of water

Temperature_April_May_eng.csv contains data on water temperature (t°C) in spawning ponds taken within a month after amphibian clutches were laid. The temperature was taken manually between the end of April and the end of May using a mercury thermometer with a scale interval 0.5 °C (standard thermometer:ТП-22, Thermopribor, Russia). The thermometer was calibrated in the factory and needed no further calibration. During measurements, the thermometer was positioned at approx. 20 cm below the surface and out of direct sunlight. All measurements were done in the morning (8–10 AM).

### Amphibian populations

Populations_status_1977_2013_eng.csv contains data regarding amphibian population dynamics between 1977 and 2013 (Data Citation 1):

1. Siberian salamander (*Salamandrella keyserlingii* Dybovski, 1870),
2. Common newt (*Lissotriton vulgaris* Linnaeus, 1758),
3. Common toad (*Bufo bufo* L., 1758),
4. Moor frog (*Rana arvalis* Nilsson, 1842),
5. Common frog (*Rana temporaria* Linnaeus, 1758),
6. Lake frog (*Pelophylax ridibundus* Pallas, 1771).

### Fecundity

**Figure 3:**
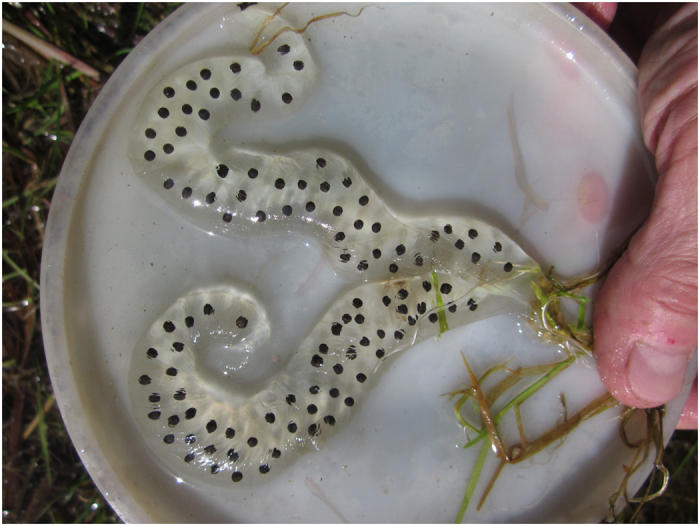
Digital image of *Salamandrella keyserlingii* spawn. Note that the spawn is spread so that every egg is visible and easy to count.

Files Spawns_number_Salamandrella_eng.csv, Spawns_number_Rana_arvalis_eng.csv, and Spawns_number_Rana_temporaria_eng.csv (Data Citation 1) contain data on the number of spawns in habitats of *S. keyserlingii, R. arvalis,* and *R. temporaria*, respectively. The number of eggs within each clutch/spawn is presented in files Fecundity_Rana_temporaria_eng.csv, Fecundity_Rana_arvalis_eng.csv, and Fecundity_Salamandrella_eng.csv (Data Citation 1). Egg clutches in every pond under investigation were counted manually. Eggs of each clutch of *Salamandrella keyserlingii* were counted separately to determine the clutch size. Originally, Prof Vershinin counted eggs in the field (at the collection site). This was necessary to avoid the transportation of the eggs to the laboratory, which could destroy the material. Starting in 2009, however, eggs were counted in the laboratory using digital images taken at the site of the collection. Importantly, preparations for egg counting were identical, with or without digital photographs, eggs being manually spread as a single-egg layer over a light plastic surface (Figure 3). Prof Vershinin took all photographs and counted eggs. The number of eggs in a clutch of brown frogs (*R. arvalis* Nilss. and *R. temporaria* L.) was calculated by sparing technique^7^. In each case, firstly, the volume of a clutch was determined with a graduated cylinder (scale interval 5 ml); secondly, the volume of 100 separated eggs was measured; thirdly, the number of eggs in a whole clutch was calculated by dividing the first number by the second. Fecundity was determined only for those three species in which it can be done *in vivo*. To ensure reproducibility and consistency of the data collection, the same laboratory equipment (chemical glasses and cylinders) has been used throughout the whole period of the observation with one exception. The following example demonstrates our approach to reduce data collection bias.

### Egg collection methods

To estimate the size of eggs in *Rana arvalis* population, we randomly select approximately 20 spawns out of 50–200 per spawning pond. From each spawn, we randomly isolate 20 eggs and place them in a container. This ensures the preservation of the material during transportation. The eggs are immediately brought to the laboratory where the size of the eggs and their developmental stage are determined under the binocular.

### Plants

Vegetation_description_eng.csv (Data Citation 1) contains data on plants found in each zone in 1977 and again in 2007. Prof Vershinin has performed the identification of dominant species of shoreline plants.

### Sample collection strategy

We used the following basic strategy in our sampling program. First of all, in spring we always collect biological material and perform on-site measurements on clear sunny days. This ensures the best visibility throughout the collection area and thus prevents losing or unintentionally destroying the material. Secondly, it is important to be able to access any spot within the collection area and, since the terrain is not always ‘user-friendly’, the collector always wears full hip boots. Thirdly, sufficient length of the collection period is very important, as spawnings in different ponds and in different seasons end at variable times in the season. The procedure begins after time when mating is finished to be sure that all of spawns from the ponds were counted. Every year (1977–2013) 55 ponds were searched by author for spawn’s number evaluation, adults and juvenile’s and water sampling. Juveniles were collected by hands near the ponds soon after metamorphose finishing. Mentioned procedures were made 4–5 times during the season (in spring, summer and autumn). So we spent about 623.1 person hours per pond. Therefore, to ensure the completeness of the data, we visit all collection sites regularly until no fresh spawns can be found. Finally, the same person, Prof Vershinin has performed the collection of all samples. Based on many years of field zoological work, this is the best approach to providing consistency of data.

## Data Records

The following files are publicly available (Data Citation 1):

1. Egg_size_Rana_arvalis_eng.csv contains data collected in 1990–1995 regarding diameter of *R. arvalis* eggs.) the data can be found in the data can be found in Egg_size_Rana_arvalis_eng.csv. Roman figures in working csv files were replaced by arab respectively: II—2, III—3, IV—4, C—5. Letters ‘NA’ indicating that surveys were not undertaken at that site in that year. Column headings:
  - 1.1. Stage: The developmental stage of the animal, based on Dabagyan and Sleptsova^8^.
  - 1.2. Diameter: The diameter of the eggs in millimeters (mm).
  - 1.3. Zone: The identifier of the urbanization degree at the sample collection site based on our landscape typification scheme.
  - 1.4. Date: The calendar date (dd.mm.yy or year only) in which the eggs were sampled.
  - 1.5. Habitat: The identifier for each wetland where the eggs were sampled.
2. Fecundity_Salamandrella_eng.csv contains data collected from 1978 to 2013 on productiveness of Siberian salamander (*Salamandrella keyserlingii,* Dybovski, 1870). Column headings:
3. 2.1. N small: The number of eggs in the smaller string of a single spawn.
4. 2.2. N Big: The number of eggs in the bigger string of a single spawn; ‘no pair’ means that only one string was found.
5. 2.3. Fecundity: Total number of eggs in a single clutch/spawn. *Salamandrella keyserlingii* spawn consists of two strings, therefore, Fecundity=N small+N Big.
6. 2.4. Zone: The identifier of the urbanization degree at the sample collection site based on our landscape typification scheme.
7. 2.5. Habitat: The identifier for each wetland where the clutch was sampled.
8. 2.6. Year: The calendar year in which the clutch was sampled.
9. Temperature_April_May_eng.csv contains data on temperatures (t°C) in spawning ponds measured during the first month from the moment when spawn clutches were laid (usually, from the end of April until the end of May); temperatures (in Celsius) were taken with thermometer (see Methods). Column headings:
10. 3.1. t°C (April-May): water temperature measured during the first month after the clutches were deposited.
11. 3.2. Zone: The identifier of the urbanization degree at the sample collection site based on our landscape typification scheme for each habitat where temperatures were taken.
12. 3.3. Year: The calendar year in which the temperature was measured.
13. 3.4. Habitat: The identifier for each wetland where the clutches were deposed.
14. Fecundity_Rana_temporaria_eng.csv contains data collected from 1978 to 2013 on productiveness of common frog (*Rana temporaria*, Linnaeus, 1758). Column headings:
15. 4.1. N: Total number of eggs in a single clutch/spawn (fecundity). Fecundity is calculated by dividing the clutch volume by volume of directly counted 100 eggs.
16. 4.2. Zone: The identifier of the urbanization degree at the sample collection site based on our landscape typification scheme.
17. 4.3. Habitat: The identifier for each wetland where the clutch was sampled.
18. 4.4. Year: The calendar year in which the clutch was sampled.
19. Populations_status_1977_2013_eng.csv contains information about presence of particular species of amphibians each year from 1977 to 2013. Each species is marked by a number: 1—*S. keyserlingii, 2—L. vulgaris, 3—B. bufo*, 4—*R. arvalis*, 5*—R. temporaria*, 6*—P. ridibundus.* Column headings:
20. 5.1. Habitat: The identifier for each wetland where amphibian species were mentioned.
21. 5.2. Zone: The identifier of the urbanization degree at the sample collection site based on our landscape typification scheme for each habitat where amphibian species were registered.
22. 5.3. Columns 1977 up to 2013 show year-by-year information on the species presence (or disappearance and habitat destruction).
23. Fecundity_Rana_arvalis_eng.csv contains data collected from 1978 to 2013 on productiveness of moor frog (*Rana arvalis,* Nilsson, 1842). Column headings:
24. 6.1. N: Total number of eggs in a single clutch/spawn (fecundity). Fecundity is calculated by dividing the clutch volume by volume of directly counted 100 eggs.
25. 6.2. Zone: The identifier of the urbanization degree at the sample collection site based on our landscape typification scheme.
26. 6.3. Habitat: The identifier for each wetland where the clutch was sampled.
27. 6.4. Year: The calendar year in which the clutch was sampled.
28. Lissotriton_juveniles_morpology_eng.csv contains data on *Lissotriton vulgaris* (Linnaeus, 1758) juveniles. Column headings:
29. 7.1. L: Individual snout-vent body length (no tail) (mm).
30. 7.2. P: Individual body weight (mg).
31. 7.3. Hep: Individual liver weight (mg).
32. 7.4. Cor: Individual heart weight (mg).
33. 7.5. Zone: The identifier of the urbanization degree at the sample collection site based on our landscape typification scheme.
34. 7.6. Habitat: The identifier for each wetland where the clutches were laid.
35. 7.7. Date: The calendar date (dd.mm.yy or year) when animals were sampled.
36. 7.8. Remarks: Special features, which include a) length of reduced piece of tail, b) presence of tail’s bud (marked by letter—‘b’), c) trace from tail’s bud (letter—‘t’), and d) anomalies.
37. Pelophylax_ridibundus_juveniles_morphology_eng.csv contains data on *Pelophylax ridibundus* (Pallas, 1771) juveniles. Column headings:
38. 8.1. L: Individual body length (mm).
39. 8.2. P: Individual body weight (mg).
40. 8.3. Hep: Individual liver weight (mg).
41. 8.4. Cor: Individual heart weight (mg).
42. 8.5. Zone: The identifier of the urbanization degree at the sample collection site based on our landscape typification scheme.
43. 8.6. Sex: Specimen sex at autopsy.
44. 8.7. Morpha: The identifier of the presence or absence of middorsal stripe (striata) based on Berger and Smielowski^9^.
45. 8.8. Habitat: The identifier for each wetland where the clutches were deposed.
46. 8.9. Date: The calendar date (dd.mm.yy or year) in which the animal was sampled.
47. 8.10. Remarks: Special features, which include a) length of reduced piece of tail, b) presence of tail’s bud (marked by letter—‘b’), c) trace from tail’s bud (letter—‘t’), and d) anomalies.
48. Rana_arvalis_ juveniles_morphology_eng.csv. The file contains data on *Rana arvalis* (Nilsson, 1842) juveniles. Column headings:
49. 9.1. L: Individual body length (mm).
50. 9.2. P: Individual body weight (mg).
51. 9.3. Hep: Individual liver weight (mg).
52. 9.4. Cor: Individual heart weight (mg).
53. 9.5. Zone: The identifier of the urbanization degree at the sample collection site based on our landscape typification scheme
54. 9.6. Sex: Specimen sex at autopsy.
55. 9.7. Morpha: The identifier of the presence or absence of middorsal stripe (striata) based on Shchupak^10^.
56. 9.8. Habitat: The identifier for each wetland where the clutches were deposed.
57. 9.9. Date: The calendar date (dd.mm.yy or year) in which the animal was sampled.
58. 9.10. Remarks: Special features, which include a) length of reduced piece of tail, b) presence of tail’s bud (marked by letter—‘b’), c) trace from tail’s bud (letter—‘t’), and d) anomalies.
59. Rana_temporaria_juv_morphology_eng.csv contains data on *Rana temporaria* (Linnaeus, 1758) juveniles. Column headings:
60. 10.1. L: Individual body length (mm).
61. 10.2. P: Individual body weight (mg).
62. 10.3. Hep: Individual liver weight (mg).
63. 10.4. Cor: Individual heart weight (mg).
64. 10.5. Zone: The identifier of the urbanization degree at the sample collection site based on our landscape typification scheme.
65. 10.6. Sex: Specimen sex at autopsy.
66. 10.7. Morpha: The identifier of the presence or absence of middorsal stripe (striata).
67. 10.8. Habitat: The identifier for each wetland where the clutches were deposed.
68. 10.9. Date: The calendar date (dd.mm.yy or year) in which the animal was sampled.
69. 10.10. Remarks: Special features, which include a) length of reduced piece of tail, b) presence of tail’s bud (marked by letter—‘b’), c) trace from tail’s bud (letter—‘t’), and d) anomalies.
70. Spawns_number_Rana_arvalis_eng.csv contains data collected from 1977 to 2013 on number of spawns in *R. arvalis* in the ponds of the different habitats. Column headings:
71. 11.1. Habitat: The identifier for each wetland where a spawn was found. Note that there are rows entitled ‘Zone II sum’, ‘Zone III sum’, or ‘Zone IV sum’ showing the sum of the spawns in zones II, III, or IV, respectively.
72. 11.2. Zone: The identifier of the urbanization degree at the sample collection site based on our landscape typification scheme for each habitat where spawns were counted.
73. 11.3. Columns 1977 to 2013 show year-by-year numbers of spawns.
74. Spawns_number_Rana_temporaria_eng.csv contains data collected from 1977 to 2013 on number of spawns in *R. temporaria* in the ponds of the different habitats. Column headings:
75. 12.1. Habitat: The identifier for each wetland where a spawn was found. Note that there are rows entitled ‘Zone II sum’, ‘Zone III sum’, or ‘Zone IV sum’ showing the sum of the spawns in zones II, III, or IV, respectively.
76. 12.2. Zone: The identifier of the urbanization degree at the sample collection site based on our landscape typification scheme for each habitat where spawns were counted.
77. 12.3. Columns 1977 to 2013 show year-by-year numbers of spawn.
78. Water_1980_2013_short_eng.csv contains data on certain chemical characteristics of water in amphibian spawning ponds. Column headings:
79. 13.1. Habitat: The identifier for each wetland where water samples were taken for chemical analysis.
80. 13.2. Date: The calendar date (dd.mm.yy or month and year) when the water sample was taken.
81. 13.3. Year: The calendar year in which the water sample was taken.
82. 13.4. t°С: The identifier of temperature of water when the sample was taken (in Celsius).
83. 13.5. Zone: The identifier of the urbanization degree at the sample collection site based on our landscape typification scheme for each habitat where the water sample was taken.
84. 13.6. NH_4_: The identifier of ammonium ions concentration (mg/dm^3^).
85. 13.7. NO_2_: The identifier of nitrites ions concentration (mg/dm^3^).
86. 13.8. NO_3_: The identifier of nitrates ions concentration (mg/dm^3^).
87. 13.9. N min: The identifier of nitrogen minimal concentration.
88. 13.10. PO_4_^3−^: The identifier of phosphates ions concentration (mg/dm^3^).
89. 13.11. Water hardness: The identifier of water hardness (the sum of calcium and magnesium ions concentration).
90. 13.12. Ca: The identifier of calcium ions concentration (mg/dm^3^).
91. 13.13. Mg: The identifier of magnesium ions concentration (mg/dm^3^).
92. 13.14. HCO_3_: The identifier of hydrocarbonate ions concentration (mg/dm^3^).
93. 13.15. Cl: The identifier of chloride ions concentration (mg/dm^3^).
94. 13.16. SO_4_: The identifier of sulfate ions concentration (mg/dm^3^).
95. 13.17. Na: The identifier of sodium ions concentration (mg/dm^3^).
96. 13.18. К: The identifier of potassium ions concentration (mg/dm^3^).
97. 13.19. Ion sum: The identifier of the concentration of all ions in a sample.
98. 13.20. Mineralization: The identifier of total mineral content (mg/dm^3^)
99. 13.21. Transparency: The identifier of water transparency (centimeters).
100. 13.22. рН: The identifier of water acidity.
101. 13.23. O_2_: The identifier of dissolved oxygen concentration (mg/dm^3^).
102. 13.24. Oxidizability: The identifier of total organic carbon content in water.
103. 13.25. BOD_5_: The identifier of biochemical oxygen demand (mg/dm^3^)—the amount of dissolved oxygen needed by aerobic biological organisms to break down organic material present in water sample over 5 days at 20 t°С.
104. 13.26. COD: The identifier of chemical oxygen demand estimated from the concentration of oxidizable compound in the sample, based on its stoichiometric reaction with oxygen to yield CO_2_ (mg/dm^3^).
105. 13.27. Coloration: The identifier of water coloration based the Platinum-Cobalt Scale.
106. Spawns_number_Salamandrella_eng.csv contains data collected from 1977 to 2013 on number of spawns in *S. keyserlingii* in the ponds of the different habitats. Since *S. keyserlingii* females normally produce 2 strings in one clutch, the data on the number of strings is also provided. Column headings:
107. 14.1. Habitat: The identifier for each wetland where a spawn was found. Note that there are rows entitled ‘Zone II sum’, ‘Zone III sum’, or ‘Zone IV sum’ showing the sum of the spawns in zones II, III, or IV, respectively.
108. 14.2. Zone: The identifier of the urbanization degree at the sample collection site based on our landscape typification scheme for each habitat where spawns were counted.
109. 14.3. Columns 1977 to 2013 show year-by-year numbers of spawn/strings.
110. Vegetation_description_eng.csv contains data on the composition of plant species community in main habitats of the amphibians. Column headings:
111. 15.1. Species: The identifier of plants found (Latin names).
112. 15.2. Habitat: The identifier for each wetland where plant communities were described.
113. 15.3. Zone: The identifier of the urbanization degree at the sample collection site based on our landscape typification scheme for each habitat where plant communities were described.
114. 15.4. Year: The identifier of calendar year in which the description has been made.
115. Habitats_coordinates.csv contains information about geographical coordinates of 26 amphibian habitats in Yekaterinburg region and the information on environmental conditions of these habitats and amphibian populations. Column headings:
116. 16.1. Habitat: The identifier for each wetland where amphibian populations were registered
117. 16.2. Zone: The identifier of the urbanization degree at the sample collection site based on our landscape typification scheme for each habitat where amphibian populations were registered
118. 16.3. Longitude (E): The identifier of longitudinal position of habitat.
119. 16.4. Latitude (N): The identifier of latitudinal position of habitat.
120. 16.5. Current status: The identifier of current existence of the population.

## Technical Validation

The authors have taken a variety of steps to ensure high data quality and the consistency of data collection methods, so that the data obtained are comparable across sites and through time. Given the longevity of the observation period, we strictly adhered to the principles of data collection, which are best described in the Smithsonian Manual^11^, an excellent textbook written by ten leading herpetologists.

All measurements were taken with factory calibrated devices under identical standardized conditions to protect against loss of precision in the measurements. Spawns were counted in clear, sunny periods just after spawning is finished, to ensure best performance of the procedure. These techniques are described in detail in the Fecundity and Eggs collection methods sections. Linear measurements were made on animals placed on horizontal, plain and solid surfaces. Damaged and abnormal eggs and individuals were excluded from analysis. The same parameters were maintained for all years of investigation. All samples were randomized, representative and independent^11^.

## Additional information

**How to cite this article:** Vershinin, V. L. *et al.* Long-term observation of amphibian populations inhabiting urban and forested areas in Yekaterinburg, Russia. *Sci. Data* 2:150018 doi: 10.1038/sdata.2015.18 (2015).

## Supplementary Material



## Figures and Tables

**Table 1:** Zone-Specific Average Temperatures (May, 1977–2013).

**Zone**	**Average t °C±s.e.m.**	***N***
II	14.7±0.28	265
III	13.5±0.25	338
IV	11.7±0.23	392
C	11.7±0.33	194

**Table 2:** Zone-Specific Average Temperatures (May, 1998–2007).

**Zone**	**Average t °C±s.e.m.**	***N***
II	13.9±0.41	102
III	13.1±0.28	230
IV	12.4±0.32	173
C	10.7±0.43	95

**Table 3:** Geographical coordinates of collection sites.

**№**	**Habitat (abbreviation)**	**Zone**	**Longitude (E)**	**Latitude (N)**	**Current status**
1	Belinskogo	II	60°37'14.63"E	56°49'9.97"N	Disappeared in 2004 (destroyed)
2	Central park	II	60°38'7.11"E	56°49'1.86"N	Disappeared in 1999 (destroyed)
3	Cherepanova	II	60°34'3.72"E	56°51'27.80"N	Disappeared in 1999 (destroyed)
4	Dekabristov	II	60°36'41.29"E	56°49'27.43"N	Still exists
5	Jasnaja	II	60°34'22.31"E	56°48'50.78"N	Still exists
6	Juzhnaja	II	60°36'41.21"E	56°47'46.62"N	Disappeared in 2012 (destroyed)
7	Krylova	II	60°34'13.31"E	56°50'36.01"N	Disappeared in 2008 (destroyed)
8	Kujbysheva	II	60°36'33.19"E	56°49'36.74"N	Disappeared in 2010 (destroyed)
9	Ol'khovka	II	60°34'38.60"E	56°51'7.88"N	Disappeared in 1993 (strongly transformed)
10	Polzunova	II	60°38'16.74"E	56°54'34.20"N	Disappeared in 1981 (destroyed)
11	Samoletnaja (front side)	II	60°38'29.29"E	56°47'39.13"N	Disappeared in 1986 (destroyed)
12	Raz’jezd	III	60°31'35.02"E	56°50'12.92"N	Still exists
13	Central park (back side)	III	60°38'39.17"E	56°48'27.96"N	Still exists
14	Kontrolnaja	III	60°32'7.19"E	56°48'58.39"N	Disappeared in 2011 (destroyed)
15	Ol'khovka (back side)	III	60°33'58.34"E	56°51'44.89"N	Disappeared in 2008 (destroyed)
16	Patrushikha	III	60°39'9.67"E	56°46'43.24"N	Still exists
17	Patrushikha (backside)	III	60°38'20.77"E	56°46'18.38"N	Still exists
18	Samoletnaja	III	60°39'3.68"E	56°47'59.35"N	Still exists
19	Elizabeth	IV	60°38'22.31"E	56°45'27.54"N	Still exists
20	Kalinovskie razrezy (Kalin.razr)	IV	60°39'10.43"E	56°54'39.75"N	Still exists
21	Obroshino	IV	60°27'22.52"E	56°53'1.44"N	Still exists
22	Shartash	IV	60°40'47.68"E	56°51'21.16"N	Still exists
23	South-West forest park	IV	60°31'31.60"E	56°48'28.28"N	Still exists
24	Shuvakish	IV	60°33'59.45"E	56°54'24.87"N	Still exists
25	Rezhevskoi trakt (Rezh.trakt)	C	60°48'53.45"E	56°58'9.10"N	Still exists
26	New Moscow trakt (N.M.trakt)	C	60°30'22.28"E	56°49'46.01"N	Still exists
